# Niche differentiation of Mucoromycotinian and Glomeromycotinian arbuscular mycorrhizal fungi along a 2-million-year soil chronosequence

**DOI:** 10.1007/s00572-023-01111-x

**Published:** 2023-05-11

**Authors:** Thomas M. Mansfield, Felipe E. Albornoz, Megan H. Ryan, Gary D. Bending, Rachel J. Standish

**Affiliations:** 1 Environmental and Conservation Sciences, Murdoch University, 90 South Street, Murdoch, WA 6150 Australia; 2 Land and Water, Commonwealth Scientific and Industrial Research Organisation, Wembley, WA Australia; 3 School of Agriculture and Environment, UWA, University of Western Australia, Crawley, WA 6009 Australia; 4 School of Life Sciences, University of Warwick, Coventry, CV4 7AL UK

**Keywords:** Fine root endophyte, FRE, MFRE, Mycorrhiza, Phosphorus-limited soils, Soil age

## Abstract

Current literature suggests ecological niche differentiation between co-occurring Mucoromycotinian arbuscular mycorrhizal fungi (M-AMF) and Glomeromycotinian AMF (G-AMF), but experimental evidence is limited. We investigated the influence of soil age, water availability (wet and dry), and plant species (native *Microlaena stipoides* and exotic *Trifolium subterraneum*) on anatomical root colonisation and DNA profiles of M-AMF and G-AMF under glasshouse conditions. We grew seedlings of each species in soils collected from the four stages of a soil chronosequence, where pH decreases from the youngest to oldest stages, and phosphorus (P) is low in the youngest and oldest, but high in the intermediate stages. We scored the percentage of root length colonised and used DNA metabarcoding to profile fungal richness and community composition associated with treatment combinations. Soil age, water availability, and plant species were important influencers of root colonisation, although no M-AMF were visible following staining of *M. stipoides* roots. Soil age and host plant influenced fungal richness and community composition. However, response to soil age, potential host species, and water availability differed between M-AMF and G-AMF. Root colonisation of *T. subterraneum* by M-AMF and G-AMF was inversely correlated with soil P level. Community composition of M-AMF and G-AMF was structured by soil age and, to a lesser extent, plant species. Richness of M-AMF and G-AMF was negatively, and positively, correlated with available P, respectively. These findings are experimental evidence of ecological niche differentiation of M-AMF and G-AMF and invite further exploration into interactive effects of abiotic and biotic factors on their communities along successional trajectories.

**Supplementary Information:**

The online version contains supplementary material available at 10.1007/s00572-023-01111-x.

## Introduction

Improvements in molecular tools have uncovered the phylogeny of Mucoromycotinian arbuscular mycorrhizal fungi (M-AMF)—previously known as “fine root endophytes” and also known as “Mucoromycotinian fine root endophytes (MFRE)”—to be distinct from the Glomeromycotinian AMF (G-AMF), among which M-AMF previously were placed taxonomically (Orchard et al. 2017a). Prior to this discovery, all studies of M-AMF were morphological (i.e., microscope observations after clearing and staining plant roots) and most studies did not distinguish M-AMF from G-AMF (Orchard et al. 2017b). Thus, little is known about the ecological niches that M-AMF occupy. However, it is apparent that both M-AMF and G-AMF widely co-occur in natural and agricultural systems (e.g., Orchard et al. 2017b; Albornoz et al. 2021, 2022), and this has opened questions about the ecological niche differentiation of these two arbuscule-forming groups of fungi.

Mucoromycotinian AMF have an ancient relationship with bryophytes (Rimington et al. 2018, 2019), which features a traditional mycorrhizal carbon (C)-for-nutrient exchange (Field et al. 2015; Hoysted et al. 2019, 2021). Furthermore, M-AMF can co-occur with G-AMF, suggesting complementary roles in bryophyte hosts (Field et al. 2016, 2019). Mucoromycotinian-AMF also colonise late-diverging vascular plants: in a meta-analysis of 108 studies, 53 plant families were found to host M-AMF, with Poaceae being the most frequently observed host family (Orchard et al. 2017b). In the late-diverging vascular plants, M-AMF again often co-occur in roots with G-AMF (Orchard et al. 2017b; Jeffery et al. 2018; Albornoz et al. 2021) and can obtain C in return for nutrients (Crush 1973; Hoysted et al. 2022). However, the body of literature on M-AMF in late-diverging vascular plants is limited compared with that on G-AMF, and additional experimental research is needed to fully understand the responses of M-AMF and G-AMF to abiotic and biotic factors and, crucially, whether those responses differ.

Ecological niche refers to the abiotic and biotic conditions under which a species can survive and reproduce (Grinnell 1924; Hutchinson 1957). If M-AMF and G-AMF are shown to favour different environmental (ecological) conditions, they may have distinct ecologies that could be important for their coexistence. Mucoromycotinian AMF have been observed within the major biomes (Orchard et al. 2017b; Albornoz et al. 2022), across both agricultural (e.g., Abbott and Robson 1978, 1982; Albornoz et al. 2022; Viscarra Rossel et al. 2022) and native ecosystems (e.g., Bueno de Mesquita et al. 2018; Postma et al. 2007; Albornoz et al. 2022). Observations within native ecosystems suggest that M-AMF occur within harsh environments such as those with low temperatures, acidic soils, and waterlogged soils (Wang et al. 1993; Orchard et al. 2017b) and can be more abundant (measured as root colonisation) than G-AMF in severe conditions, particularly within cold climates (Crush 1973; Blaschke 1991; Olsson et al. 2004; Newsham et al. 2017), and under both waterlogged (Orchard et al. 2016), and drought conditions (Staddon et al. 2004). Preferences of M-AMF for both waterlogged and drought conditions could suggest a broad ecological niche with regards to water availability. Alternatively, M-AMF may show niche variation among taxa, with different taxa possibly being well suited to different levels of water availability, which together sum to a broad ecological niche across multiple M-AMF. This broad ecological niche is also apparent for G-AMF in regard to water availability, as they can show a high root colonisation in an extremely high precipitation (~ 3000–4000 mm mean annual rainfall) tropical ecosystems (e.g., Fischer et al. 1994; Gehring and Connel 2006), but also can show a low root colonisation in waterlogged pastures (Orchard et al. 2016) and be suppressed by a high precipitation (> 650 mm mean annual rainfall) in savannah ecosystems (Stevens et al. 2020). A preference for particular host plant species also may cause niche differentiation of M-AMF and G-AMF. Ryan and Kirkegaard (2012) found M-AMF to occupy a greater proportion of root length colonised by AMF for wheat (*Triticum aestivum* L.) than for field pea (*Pisum sativa* L.). Few studies have explored the interactive effects of the above-mentioned factors on niche dimensions of M-AMF and G-AMF.

In Australia, Albornoz et al. (2022) surveyed paired native vegetation and farm sites across the continent and found a strong preference by M-AMF for temperate biomes, whilst G-AMF were abundant in temperate through to the tropical biomes. Albornoz et al. (2022) also found that while sequence abundance of M-AMF was the highest in arid farmlands, richness was the highest in cold and wet temperate biomes, such as montane forests and grasslands. Mucoromycotinian AMF also exhibited a preference for agricultural over native systems, whereas G-AMF showed no preference (Albornoz et al. 2022). This could suggest that M-AMF strongly associate with agricultural plants (often exotic annual grasses and legumes), in contrast to the apparent lack of strong host preference by G-AMF suggested by their wide host range (Brundrett and Tedersoo 2018). Within temperate pastures of southern Australia, dominated by the exotic pasture legume *Trifolium subterraneum*, Albornoz et al. (2021) found that M-AMF and G-AMF exhibited ecological niche differentiation driven by factors including soil characters, temperature, and rainfall.

Here, to investigate further the ecological niche of M-AMF and G-AMF in Australian native ecosystems, we utilised a soil chronosequence as the basis for a manipulative experiment. Soil chronosequences are different aged sequences of soils of the same origin (Lambers et al. 2017); changes in soil nitrogen (N), phosphorus (P), and pH reflect stages of long-term soil development (Laliberté et al. 2012; Table 1). In most Australian retrogressive soil chronosequences, N is often extremely scarce in young soils until it is increased by nitrogen-fixing plants in intermediate soils, then steadily declines as soils age, whereas P concentration often begins high and leaches as soils age (Walker and Syers 1976; Turner et al. 2018; Table 1). Lastly, soils acidify as they age, and alkaline or neutral soils can become acidic in the oldest soils of the chronosequence (Tang and Rengel 2003; Turner et al. 2018; Table 1). Consequently, soil chronosequences offer an ideal study system to evaluate the influence of soil conditions on M-AMF and G-AMF while holding constant the other factors (e.g., dispersal) that could affect distribution and abundance of the fungi.

**Table 1:** Soil properties of the Warren chronosequence within each chronosequence stage (soil age) of the study summarised from Turner et al. (2018). A subset of the soil ages from Turner et al. (2018) was used

Chronosequence stage	1	3	4	6
Soil age	< 6.5 ka	~ 6.5 ka	120–500 ka	> 2,000 ka
pH (CaCl_2_)	***8.1***	5.3	4.9	4.0
TN (g m^−2^)	88	***239.5***	99.4	74.1
TP (g m^−2^)	***35.6***	18.4	11.4	2.3
Resin P (mg kg^−1^)	~ 3	** ~ *8***	~ 5	~ 2

Bolded and italicized values are the highest levels among the sampled stages

*TN* total nitrogen, *TP* total phosphorus (both from the top 100 cm of the soil profile), *Resin P* readily available phosphorus (top 10 cm of the soil profile)

Along retrogressive soil chronosequences, as soils age and soil properties change, abundance of G-AMF declines (Balser et al. 2005; Welc et al. 2012; Teste et al. 2016) although responses of richness and composition remain unresolved. Plant communities changing with soil age also can influence communities of G-AMF (Martínez-García et al. 2015); however, declines in abundance of G-AMF are most likely due to an extremely low fertility in the oldest stages of soil chronosequences (i.e., oldest soils; Turner et al. 2018). Although the general consensus is that abundance of G-AMF increases with decreasing soil P (Smith and Read 2010), there is a point at which P becomes so scarce that it limits G-AMF as much as it does the plant host (Bolan et al. 1987; Jeffery et al. 2017). The same has been shown for M-AMF (Jeffery et al. 2018), indicating that the abundance of both M-AMF and G-AMF can be hindered at both extremely low and high levels of P.

Shifts in soil pH along soil chronosequences also may influence abundances, as M-AMF tend to prefer acidic soils, whereas many species of G-AMF do not (Wilson and Trinick 1983; Wang et al. 1985; Postma et al. 2007). Soil pH is controlled by multiple chemical processes in soils responsible for releasing H + ions including leaching, mineralisation, and nitrification (Neina 2019). In turn, pH controls the soil biological community and its biological processes (Neina 2019), making it difficult to pin-point an exact mechanism for soil pH influencing AMF. Changes in soil physicochemical properties not only affect abundance but also richness and community composition of these fungi (e.g., Balser et al. 2005; Göransson et al. 2008; Davison et al. 2021). Richness of G-AMF declines with increasing soil pH (Albornoz et al. 2021). However, soil pH can have both positive (Albornoz et al. 2022) and negative effects (Albornoz et al. 2021) on richness of M-AMF. Hence, it is likely that communities of both M-AMF and G-AMF change during ecosystem development, but the extent to which this response differs between M-AMF and G-AMF remains to be determined.

Mucoromycotinian-AMF and G-AMF could also differ along a chronosequence because of different host plant preferences. Along long-term soil chronosequences, the youngest stages are the highly N-limiting, with most nutrients available in young-intermediate stages, which then become progressively P-limiting with age (Laliberté et al. 2012). Plant biomass follows the same trend, peaking in young-intermediate stages and trending lower in the youngest and old stages (Peltzer et al. 2010). Furthermore, nutrient declines result in shifts in plant communities and nutrient acquisition strategies, including relative dominance of non-mycorrhizal strategies at low P (Zemunik et al. 2015, 2016; Lambers et al. 2017). Communities of G-AMF shift with host plant communities along soil chronosequences (Martínez-García et al. 2015; Dickie et al. 2013); it is unknown if M-AMF communities change similarly.

Here, we used a manipulative experiment to compare the influences of soil age, water availability, and host identity on anatomical root colonisation, and richness and community composition (based on DNA sequences) of M-AMF and G-AMF. To do so, we conducted a glasshouse experiment using soils of differing ages and chemistry collected from the 2-million-year-old Warren soil chronosequence in south-western Australia (Turner et al. 2018). Since the 1970s, this region has been affected by a drying climate. Annual rainfall has declined by 15–35% (Hennessy et al. 1999; Timbal et al. 2006; Nicholls 2010), and this is predicted to continue (Hope et al. 2015; Dey et al. 2019), which may have affect AMF. So, we manipulated water availability as part of our experiment design. We used a two host species, a native grass, *Microlaena stipoides* (Labill.) R.Br., and an exotic pasture legume, *Trifolium subterraneum*, and had a two levels of water availability. We hypothesised that:

1. With increasing soil age, the subsequent decline in soil pH moderated by available soil P will be associated with an increase in anatomical root colonisation by M-AMF, while that of G-AMF will decrease (Postma et al. 2007), consistent with ecological niche differentiation between the two groups of AMF.
2. Soil age will differentially drive changes in the community composition and richness of M-AMF and G-AMF based on DNA profiles of fungi in plant roots, consistent with changes in soil P (Krüger et al. 2015).
3. Anatomical root colonisation by G-AMF and M-AMF will differ in response to soil water availability and host plant species.

## Materials and methods

### Soil collection

Soils were collected from the four locations along the Warren chronosequence (− 24.62°S, 115.90°E) in the south-west of Western Australia. This area experiences average yearly temperature ranges of 10–20 °C (Bureau of Meteorology 2019) as characteristic for a temperate region (Beard 1990). The average annual rainfall from 1941 to 2019 was 1187.2 mm (Bureau of Meteorology 2019). However, within the south-west of Australia, there has been evidence of decreasing rainfall since 1910 (Haylock and Nicholls 2000; Li et al. 2005). The soil chronosequence is a complex soil system and follows the classical long-term ecosystem development model of limiting nitrogen (N) in the young stages and limiting P in the oldest stages (Laliberté et al. 2012; Turner et al. 2018). These soils are severely P-impoverished, and readily available P (resin P) and total N follow a hump-shaped pattern, being the highest within the intermediate stages (Table 1). Additionally, soil pH gradually decreases along the chronosequence (Turner et al. 2018; Table 1). The Warren ecosystem is considered to be a retrogressive soil chronosequence because soil nutrients and plant biomass decline as soils age due to pedogenesis (Peltzer et al. 2010; Turner et al. 2018).

The sampling locations were chosen to represent the four stages of the chronosequence with sampling locations spaced 1.5–2 km apart (after Turner et al. 2018). Stage 1: Meerup Unstable Sand (young) < 6.5 ka. Stage 3: Meerup Podzols over Calcareous Sands (medium) ~ 6.5 ka. Stage 4: Meerup Podzols in Siliceous Sands (old) 120–500 ka. Stage 6: Cleave (very old) > 2000 ka (Table 1). The native Warren vegetation ranges from mixed-coastal heath (Stage 1) to peppermint tree (*Agonis flexuosa* (Willd.) Sweet) overstory (Stage 3 and Stage 4) and *Banksia* mid-story dominated communities (Stage 6; Fig. S1).

In April 2019, we established two 10-m transects and collected 10 soil samples, 1 m apart, along each transect at each sampling location. Soils were sampled beneath native vegetation. Each sample was 1.2 L, removed from the 0–30 cm soil layer and bagged to form a composite sample per location. Later, soils belonging to the same chronosequence stage were combined, dried at 40 °C for 2 days, sieved using a 2-mm sieve, and thoroughly mixed. These soils were used as inoculum for the glasshouse experiment.

### Seed germination

We purchased *M. stipoides* seeds (~ 4.0 g) from the Native Seeds Pty Ltd Australia (nativeseeds.com.au, 3739 Great Alpine Rd, Eurobin, Victoria, Australia, last accessed 10/04/19), and obtained *T. subterraneum* seeds (~ 9.5 g) from stores held at the University of Western Australia, sourced from experimental plots in Perth, Western Australia. One week prior to the start of the experiment, 200 seeds of each species were surface sterilised and germinated on moist filter paper in a Petri dish. Surface sterilisation was done via soaking (10 min for *M. stipoides*, 2 min for *T. subterraneum* reflecting their seed sizes) in sodium hypochlorite (4% available chlorine), rinsing five times in sterile water, and soaking in sterile water for 60 min. *Trifolium subterraneum* seeds were germinated 3 days after the *M. stipoides* seeds so seedlings would be of the same age at planting.

### Experimental design

A multifactorial design assessed the influence of the three factors, and their interactions, on root colonisation, richness, and community composition of M-AMF and G-AMF—plant hosts (*M. stipoides* and *T. subterraneum*), water availability (wet and dry), and chronosequence stage (Stages 1, 3, 4, 6 of the Warren chronosequence). There were five replicates per treatment combination for a total of 80 pots at the beginning of the experiment.

For all treatments, 1.1 kg of dry soil was placed into 18-cm tall, 8 cm × 8-cm wide, 1-L sealed plastic pots. Each pot was watered to 100% field capacity (FC; measured gravimetrically) with deionised (DI) water and three seedlings of the same plant species were sown per pot. Plastic beads were added to cover the soil (~ 20 g) to prevent excess evaporation. No fertiliser was added. Pots were placed in a random block design onto two benches within a glasshouse and remained in the same place throughout the experiment. Glasshouse air temperature was controlled at an average temperature of 20 °C. All pots were watered to 80% FC for 4 weeks, two times per week, to facilitate root colonisation by M-AMF and G-AMF and to assist establishment of the host plants. At week 4, seedlings were culled to two per pot and the water availability treatment was randomly imposed: 60–80% FC for wet and 15–35% FC for dry treatments, respectively, watering two times per week. Plants were then grown for an additional 6 weeks before harvest, when all pots had two plants.

### Experimental harvest and response variables

At harvest, composites of plant roots from each pot were washed with DI water to remove soil, cut into 1-cm pieces, homogenised, and divided into three subsamples: (1) ~ 400 mg of fine roots stored in 70% (v/v) ethanol to be assessed microscopically for root colonisation by M-AMF and G-AMF, (2) ~ 100 mg of fresh fine roots stored at − 80 °C pending DNA extraction, and (3) remaining root material (if any) which was weighed and dried at 40 °C for 5 days to calculate root water content. Shoots were removed, dried at 40 °C for 5 days and weighed.

The roots stored in ethanol were cleared in 10% KOH for 5 days at room temperature, then rinsed with 1% HCl, stained for 1 h in a 5% ink-vinegar (Parker Quink blue-black ink) solution, and de-stained in acidified glycerol for at least 24 h before assessment of root colonisation (Giovannetti and Mosse 1980; Orchard et al. 2017c).

To assess colonisation, ~ 1-cm root pieces were mounted onto slides as described by Orchard et al. (2017c). The percentage of root length colonised by AMF was determined using the line intercept method (Giovannetti and Mosse 1980) under magnifications of × 100 and × 400 until a minimum of 100 intercepts were counted. At each intercept, the presence/absence of M-AMF and G-AMF was separately recorded with the two groups distinguished by the morphology of their entry points, hyphae, and vesicles (Fig. S2). In some cases, M-AMF and G-AMF were found at the same intercept but scored separately.

### DNA extraction, amplification, and sequencing

Roots intended for DNA extraction were freeze-dried for 4 days. DNA was extracted from ~ 20 mg of dry material at the University of Western Australia using the DNeasy PowerPlant Pro Kit (50) (Qiagen, Carlsbad, USA) following the manufacturer’s protocol. The DNA amplification was performed using the primers AMV4.5NF and AMDGR that target both Mucoromycotina and Glomeromycotina sequences (Sato et al. 2005; Orchard et al. 2017a). Polymerase chain reactions (PCR) were performed in a 25-μl reaction volume, comprising the Q5® Hot Start High-Fidelity 2X Master Mix (New England Biolabs, South Hamilton, USA) and 0.5 µM of both primers. Thermocycling subjected an initial denaturation at 98 °C for 30 s followed by 35 cycles of 98 °C for 10 s, 60 °C for 15 s, and 72 °C for 20 s, and finally at 72 °C for 5 min. After PCR, the DNA amplicon was purified using the Agencourt AMPure XP beads (Beckman Coulter, Pasadena, USA) following the manufacturer’s instructions. Indices and Illumina sequencing adapters were attached to the amplicon for modification, using the Nextera XT Index Kit v2 by PCR as described in the manufacturer’s protocol. The DNA amplicons were purified and normalised using the SequalPrep™ Normalization Plate (96) Kit (Invitrogen, Carlsbad, USA) and then quantitatively assessed using a Qubit 2.0 Fluorometer (Thermo Fisher Scientific, Waltham, USA). The resulting concentration of the library was 4 nM, which was then sequenced using the MiSeq Reagent Kit v3 600-cycle (Illumina, San Diego, USA) at the University of Warwick.

For bioinformatic analysis, sequences were demultiplexed, adapter and primer sequences removed, and raw pair-ended sequences were quality checked using the *cutadapt* (Martin 2011). Sequences were clustered at a 97% identity threshold using the VSEARCH (Rognes et al. 2016) into operational taxonomic units (OTUs). At the same time, chimeras were removed de novo and OTUs with fewer than 10 sequences were removed. Consensus sequences of each OTU were subsequently queried against the SILVA database v137 (Quast et al. 2013) at 95% identity using VSEARCH (Rognes et al. 2016). We classified as M-AMF any sequence that matched to known M-AMF sequences from the Endogonaceae (Mucoromycotina subphylum) (Orchard et al. 2017a; Walker et al. 2018). We classified as G-AMF any sequence that best matched to Glomeromycotina reference sequences. Any sequences that did not match M-AMF or G-AMF were removed and not further analysed.

### Statistical analysis

The entire data set was rarefied to the smallest sequencing depth (i.e., 6282 sequences) with the ‘rarefy’ function with 10 iterations in the *vegan* package (Oksanen et al. 2015), as next-generation sequencing is sensitive to differing numbers of sequences among samples and rarefaction standardises to account for this difference (Dickie 2010). Finally, rarefied OTU richness was calculated for both M-AMF and G-AMF and averaged. Rarefied OTU richness is hereafter referred to as richness. We used the linear mixed effect models (Zuur et al. 2009) to test for differences in root colonisation and richness among fixed variables of chronosequence stage, water availability, host species, and their interaction using the ‘lme’ function in the *nlme* package (Pinheiro et al. 2017). Plant age at harvest was included as a random effect in all models since harvest took place over 4 days. Root dry weight was also included as a random effect for root colonisation models as this may have influenced root colonisation; however, it was not found to be influential and was dropped from subsequent models. The best models had residuals visually assessed using the qqplots for violations of model assumptions (i.e., normality and homogeneity) (Zuur et al. 2009). If assumptions were violated, new variance structures were fixed or outliers were removed, and model selection was run again. Marginal and conditional *R*^2^ were the same in all cases that we report one *R*^2^. In the case of a significant interaction among any of the explanatory variables, post hoc Tukey HSD tests were conducted using the ‘glth’ function in the *multcomp* package (Hothorn et al. 2016). The Pearson’s correlation coefficient (R Core Team 2018) was calculated to test for correlation between G-AMF and M-AMF root colonisation.

Non-metric multidimensional scaling (NMDS) was used to visualise differences in fungal community composition among treatment combinations using the Bray–Curtis dissimilarity distance metric. To test for differences in community composition among treatments, we used the permutational multivariate analysis of variables (PERMANOVA) with ‘adonis2’ function in the *vegan* package (Oksanen et al. 2015), and where appropriate, pairwise Holm comparison with adjusted *p*-values for multiple comparisons using the ‘p-adjust’ function in base R (Holm 1979). Within-group variance between soil ages was tested using the ‘betadisper’ function in the *vegan* package (Oksanen et al. 2015). All statistical analyses were conducted using the R statistical software version 4.0.3 (R Core Team 2018).

## Results

### Sequencing overview

Several pots from the dry treatment had no surviving seedlings at the end of the experiment, leaving two to five replicates per treatment combination at the end of the experiment (74 samples in total). From the 433,795 sequences obtained, 13.5% (i.e., 58,148) were M-AMF, 63.5% (i.e., 275,272) were G-AMF, while the rest were other organisms. A total of 21 OTUs of M-AMF (Endogonaceae) were found. The most abundant OTUs (i.e., OTU 6 and OTU 66; uncultured Endogonaceae) formed 63.84% and 23.22%, respectively, of the total M-AMF sequences. OTU 6 was found primarily in Stage 1, while OTU 66 was most abundant in Stage 6 but not present in Stage 1. Only 13 M-AMF OTUs were found within *M. stipoides*, while all 21 OTUs were found in *T. subterraneum* (eight OTUs unique to this host).

A total of 79 OTUs of G-AMF were found, with the most abundant ones (i.e., OTU 2 and OTU 1), forming 14.37% and 12.85%, respectively, of the total G-AMF sequences. OTU 2 belonged to the family Acaulosporaceae (*Acaulospora sp. MIB 8822*) and was primarily found within Stage 6. As per indicator species analysis; OTU 1 belonged to the family Gigasporaceae (*Scutellospora calospora*) and had high abundance in Stages 3, 4, and 6 but did not occur in Stage 1. *Microlaena stipoides* hosted 74 OTUs and *T. subterraneum* hosted 73 OTUs (six and five OTUs unique to the hosts, respectively).

### Fungal anatomical root colonisation

**Fig. 1:**
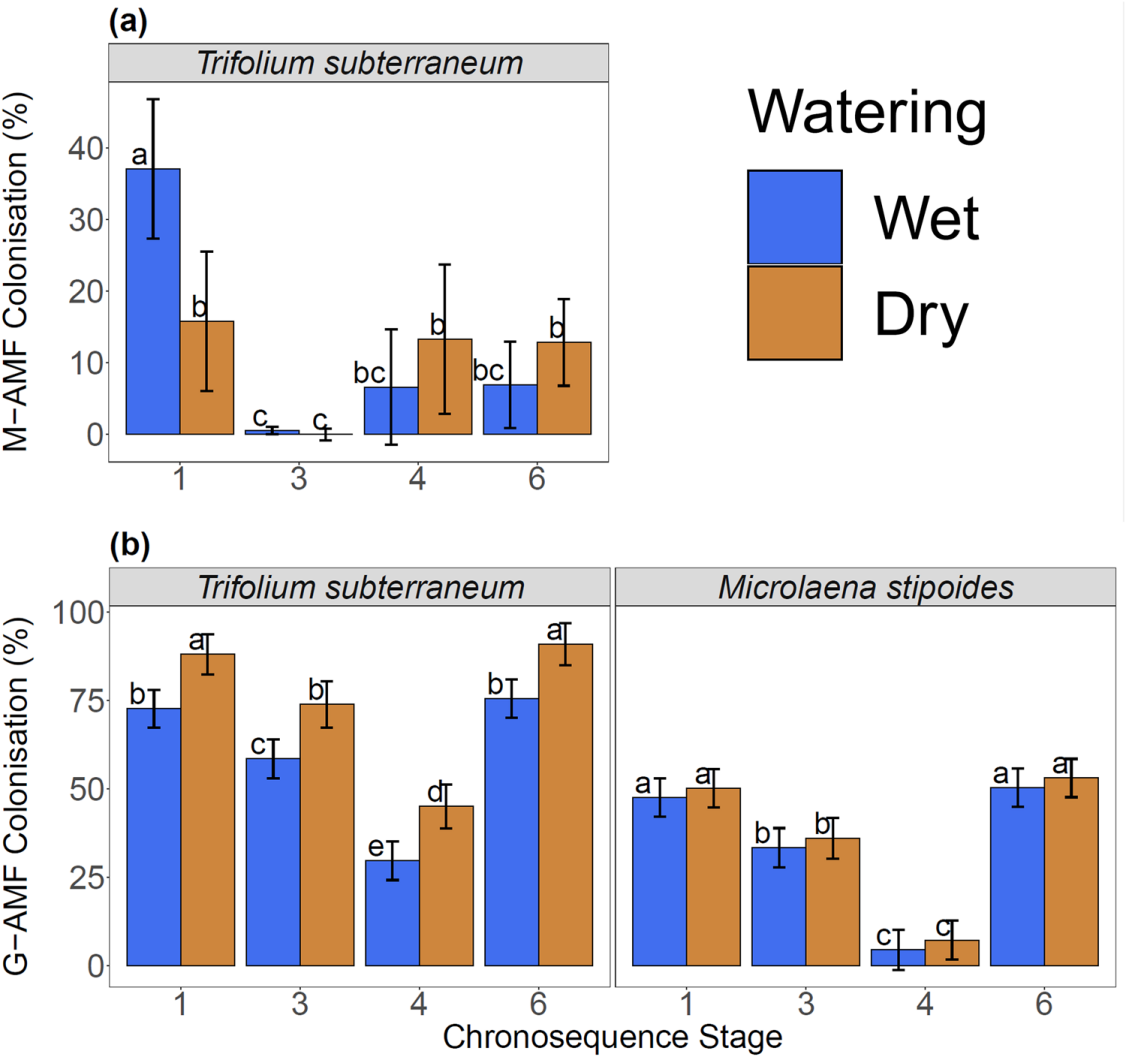
The percentage of root length colonised observed microscopically (model estimate ± 95% confidence intervals) by Mucoromycotinian arbuscular mycorrhizal fungi (M-AMF) (**a**) and Glomeromycotinian arbuscular mycorrhizal fungi (G-AMF) (**b**). Note the different scales for panels **a** and **b**. Two watering treatments, 60–80% field capacity (wet) and 15–35% field capacity (dry), were applied to each chronosequence stage (Stage 1 is the youngest, increasing to Stage 6 as oldest). Colonisation by M-AMF was not observed within *M. stipoides*. Bars topped by the same letter do not differ significantly among chronosequence stages and watering treatments (*P* < 0.05)

Anatomical root colonisation by M-AMF was found only in *T. subterraneum*, while both plant species were colonised by G-AMF. Colonisation of *T. subterraneum* roots by M-AMF was lower than that by G-AMF across all chronosequence stages and water availabilities (F_3,16_ = 17.59, *R*^2^ = 0.74, *P* < 0.001; Fig. 1). Root colonisation by M-AMF was observed in 28 of the 35 T*. subterraneum* root samples with a range of 1.0–58.5% of root length colonised, while G-AMF were observed in all samples of both hosts with 13.5–90.7% in *T. subterraneum* and 0.7–70.4% in *M. stipoides* of root length colonised. No significant correlation was found between the percentage of root length colonised by M-AMF and G-AMF (*r*_33_ = 0.14, *P* = 0.552).

Anatomical root colonisation by M-AMF of *T. subterraneum* was influenced by an interaction between chronosequence stage and water availability (Table 2). Water availability did not influence root colonisation by M-AMF, except in Stage 1, where it was twice as high in the wet than in the dry treatment (Fig. 1a). In the wet treatment, root colonisation by M-AMF was the highest in Stage 1 (Fig. 1a). In the dry treatment, root colonisation by M-AMF was the lowest in Stage 3 with no differences among the other chronosequence stages (Fig. 1a).

**Table 2:** Summary table of linear mixed effect model of anatomical root colonisation by Mucoromycotinian arbuscular mycorrhizal fungi (M-AMF) and Glomeromycotinian AMF (G-AMF) using “lme” function in *nlme* (Pinheiro et al. 2017)

	***Df***	***χ***^**2**^	***P*-value**	***R***^**2**^
**M-AMF**				**0.66**
Soil	3	42.3	** < 0.0001**	
Host	-	-	-	
Water	1	6.4	**0.011**	
Soil: host	-	-	-	
Soil: water	3	8.4	**0.037**	
Host: water	-	-	-	
Soil: host: water	-	-	-	
**G-AMF**				**0.89**
Soil	3	310.2	** < 0.0001**	
Host	1	227.3	** < 0.0001**	
Water	1	18.1	** < 0.0001**	
Soil: host	3	3.8	0.279	
Soil: water	3	5.6	0.131	
Host: water	1	10.0	**0.002**	
Soil: host: water	3	7.8	0.051	

Significant effects are in bold (*P* < 0.05). Marginal and conditional *R*^2^ did not differ

Anatomical root colonisation by G-AMF was influenced by chronosequence stage, host, water availability, and the interaction between host and water availability (Table 2). Root colonisation by G-AMF was consistently higher in *T. subteranneum* than in *M. stipoides* across all chronosequence stages and water availabilities (Fig. 1b). Both host species showed similar trends of the highest root colonisation by G-AMF within Stages 1 and 6, and the lowest in Stage 4. Root colonisation by G-AMF was higher in the dry than the wet treatment across all chronosequence stages, but only in *T. subteranneum* (Fig. 1b).

### Fungal richness

**Fig. 2:**
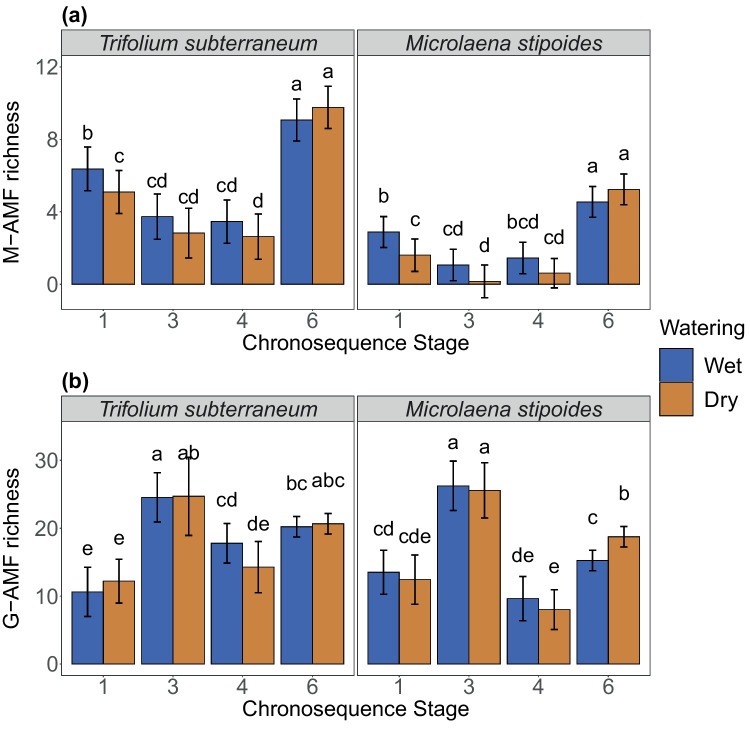
Richness (model estimate ± 95% confidence intervals) observed in DNA sequences of Mucoromycotinian arbuscular mycorrhizal fungi (M-AMF) (**a**) and Glomeromycotinian arbuscular mycorrhizal fungi (G-AMF) (**b**) in two host plant species. Note the different scales for panels **a** and **b**. Two watering treatments, 60–80% field capacity (wet) and 15–35% field capacity (dry), were applied to each chronosequence stage (Stage 1 is the youngest, increasing to Stage 6 as oldest). Bars topped by the same letter do not differ significantly among chronosequence stages and watering treatments (*P* < 0.05)

DNA-sequences of M-AMF were found in *M. stipoides* root samples, despite no anatomical root colonisation by M-AMF being observed under the microscope. Richness of M-AMF was influenced by chronosequence stage, host, and the interaction between chronosequence stage and host (Fig. 2a; Table 3). Richness of M-AMF was twice as high for *T. subterraneum* as for *M. stipoides* at each chronosequence stage and water availability combination (Fig. 2a). In both host species, M-AMF richness was approximately three times higher in Stage 6 than in Stages 3 and 4, and Stage 1 showed an intermediate M-AMF richness (Fig. 2a). Water treatment did not influence richness of M-AMF (Fig. 2a; Table 3).

**Table 3:** Summary table of linear mixed effect model of DNA sequence richness of Mucoromycotinian arbuscular mycorrhizal fungi (M-AMF) and Glomeromycotinian AMF (G-AMF)

	***Df***	***χ***^**2**^	***P*-value**	***R***^**2**^
**M-AMF**				**0.83**
Soil	3	16.0	**0.001**	
Host	1	22.5	** < 0.0001**	
Water	1	1.8	0.178	
Soil: host	3	14.8	**0.002**	
Soil: water	3	5.3	0.144	
Host: water	1	0.7	0.397	
Soil: host: water	3	4.7	0.193	
**G-AMF**				**0.92**
Soil	3	41.7	** < 0.0001**	
Host	1	1.4	0.242	
Water	1	0.2	0.658	
Soil: host	3	14.1	**0.002**	
Soil: water	3	9.0	**0.029**	
Host: water	1	0.6	0.445	
Soil: host: water	3	2.8	0.424	

Significant effects are in bold (*P* < 0.05). Marginal and conditional *R*^2^ did not differ

Richness of G-AMF was driven by the interaction between chronosequence stage and both host and water treatment (Table 3). Richness of G-AMF was the highest in Stage 3 and the lowest in Stages 1 and 4 (Fig. 2b). No differences in richness of G-AMF were found between hosts, except in Stage 4, where it was higher in *T. subterraneum* than in *M. stipoides* (Fig. 2b). Similarly, no differences in richness of G-AMF were found between water treatments, except in Stage 6 of *M. stipoides*, where it was slightly higher in the dry than the wet treatment (Fig. 2b).

### Fungal community composition

**Fig. 3:**
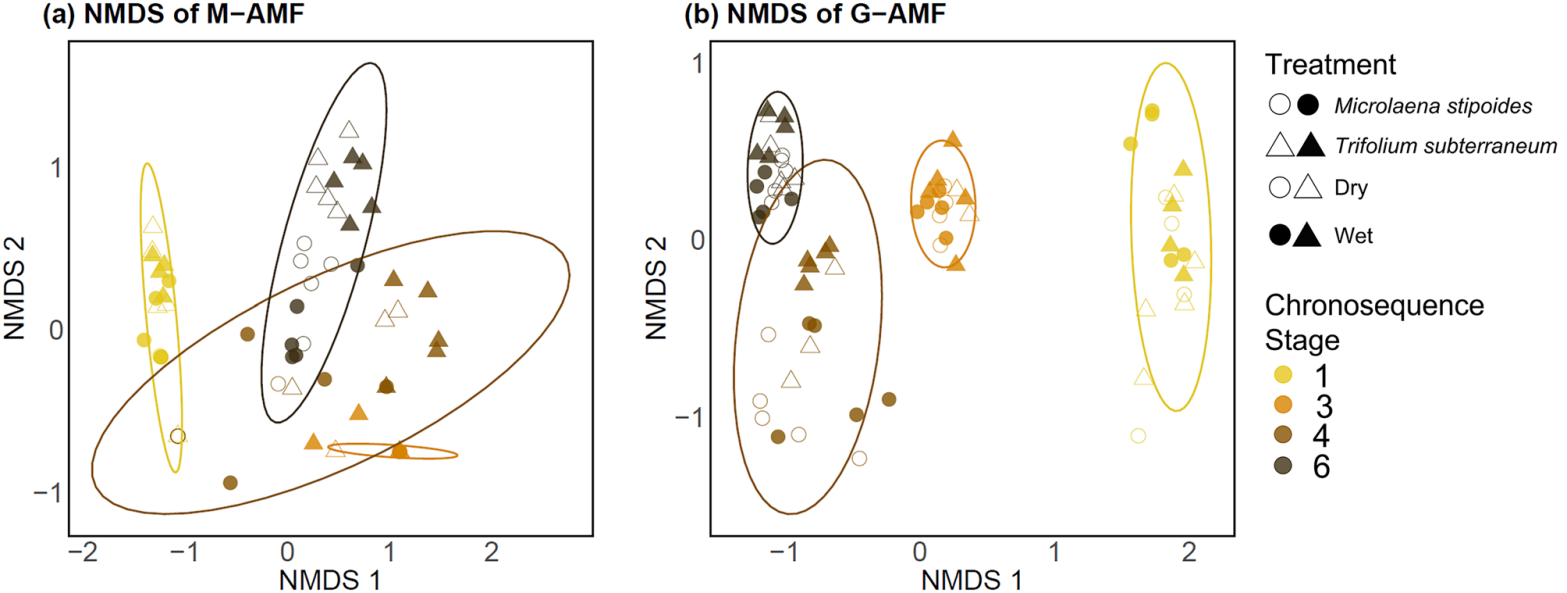
Nonmetric multidimensional scaling (NMDS) plots representing Mucoromycotinian arbuscular mycorrhizal fungi (M-AMF; stress = 0.14) (**a**) and Glomeromycotinian arbuscular mycorrhizal fungi (G-AMF; stress = 0.08) (**b**) community assemblages based on fungal DNA sampled from plant roots of each chronosequence stage. Symbols represent the two host plants; *Microlaena stipoides* (circles) and *Trifolium subterraneum* (triangles). Open symbols represent watering treatments: 60–80% field capacity (wet) and 15–35% field capacity (dry). Ellipses show 95% confidence intervals from the mean centroid within each chronosequence stage based on Bray Curtis dissimilarity scores

Non-metric multidimensional scaling ordination of M-AMF showed differences in community composition, estimated using root DNA sequence profiles, among treatment combinations (Fig. 3a). Results of the PERMANOVA revealed that community composition of M-AMF was influenced by the interaction between chronosequence stage and host species (Table 4), and between chronosequence stage and water treatment (Table 4). Chronosequence stage explained 42% of the total variation in communities of M-AMF, while host and water treatment explained 6% and 1%, respectively (Table 4). Within group variation of M-AMF was not homogenous between stages (*F* = 8.88, *P* < 0.001). Communities differed between hosts in all stages except Stage 3 (*P* = 0.07; Fig. 3a), and also differed between water treatments in the two oldest Stages 4 and 6 (*P* < 0.05 and *P* < 0.01, respectively; Fig. 3a). Stages 1 and 6 had unique communities relative to all other stages, while Stages 3 and 4 showed no difference between their communities (Fig. 3a; Table 4).

**Table 4:** Summary table of the PERMANOVA on communities of Mucoromycotinian arbuscular mycorrhizal fungi (M-AMF) and Glomeromycotinian AMF (G-AMF) using the ‘adonis2’ function in *vegan* (Oksanen et al. 2015)

	***Df***	***SumsofSqs***	***R***^**2**^	***pseudo-F***	***P*-value**
**M-AMF**					
Soil	3	7.81	0.42	20.49	**0.0001**
Host	1	1.14	0.06	8.98	**0.0001**
Water	1	0.25	0.01	1.97	0.079
Soil: host	3	2.00	0.11	5.24	**0.0001**
Soil: water	3	0.84	0.05	2.19	**0.009**
Host: water	1	0.19	0.01	1.47	0.189
Soil: host: water	3	0.32	0.02	0.85	0.608
**G-AMF**					
Soil	3	14.62	0.70	692.23	**0.0001**
Host	1	0.27	0.01	3.85	**0.006**
Water	1	0.19	0.01	2.63	**0.033**
Soil: host	3	1.09	0.05	5.17	**0.0001**
Soil: water	3	0.41	0.02	1.96	**0.027**
Host: water	1	0.10	0.01	1.37	0.214
Soil: host: water	3	3.94	0.19	1.41	0.153

Significant effects are in bold (*P* < 0.05)

The non-metric multidimensional scaling plot of G-AMF showed distinct communities primarily along NMDS axis 1 (Fig. 3b). The same interactions that affected M-AMF, chronosequence stage and host species and chronosequence stage and water treatment, affected G-AMF (Table 4). Chronosequence stage explained 70% of the total variation in communities of G-AMF, while host and water treatment explained 1% each (Table 4). There were different communities between hosts in all stages (Fig. 3b), although, communities only differed between water treatments in Stage 4 (Fig. 3b). Within group variation of G-AMF was not homogenous between stages (*F* = 6.01, *P* = 0.001). In contrast to M-AMF, communities of G-AMF were different in all pairwise comparisons between chronosequence stages (Fig. 3b; Table 4).

## Discussion

Overall, our results show that anatomical root colonisation, DNA richness, and DNA community composition of M-AMF and G-AMF were mostly driven by soil age, which is likely reflective of differences in soil P measured by Turner et al (2018). Our first hypothesis that anatomical root colonisation by M-AMF would increase with soil age and subsequent declining soil pH, while colonisation by G-AMF would decline, was not supported. Instead, we observed that root colonisation by both M-AMF and G-AMF was generally inversely proportional to available P across the chronosequence. There was partial support for our second hypothesis that fungal richness would follow available P trends across the chronosequence. This was true for G-AMF, which had the highest species richness in Stage 3, the stage with the highest P availability. The opposite was found for M-AMF, which had the lowest fungus species richness in Stage 3. Soil age strongly influenced fungal communities, partially supporting our second hypothesis. Our third hypothesis that root colonisation by M-AMF and G-AMF would differ in response to water availability also was partially supported. Root colonisation by G-AMF was higher in the dry treatment of all stages for *T. subterraneum*, while that of M-AMF was higher in the wet treatment, but only in Stage 1. Finally, we saw a low root colonisation by G-AMF and no root colonisation by M-AMF in *M. stipoides* compared to a high root colonisation by both fungal groups in *T. subterranean*.

### Influences of chronosequence stage, host, and water availability on anatomical root colonisation

Soil age was the main determinant of root colonisation by both M-AMF and G-AMF. The chronosequence stages differ with respect to vegetation and soils, including a decrease in soil pH as soil ages, and a hump-shaped trend for available P and total N (Turner et al. 2018; Table 1). Both M-AMF and G-AMF showed the highest levels of colonisation in the youngest and oldest soils, and the lowest in the middle-aged soils, indicating potential niche similarities in root colonisation. Colonisation trends of M-AMF and G-AMF did not follow the steady decline in pH along the soil chronosequence, suggesting that pH had little influence on root colonisation in this study. This was unexpected as low pH can have a strong positive influence on root colonisation by M-AMF (Postma et al. 2007; Göransson et al. 2008). Both M-AMF and G-AMF showed low colonisation in stages with the highest soil P availability, which is consistent with previous observations (Wang et al. 2017; Bueno de Mesquita et al. 2018; Albornoz et al. 2021). Additionally, while other studies have shown both M-AMF and G-AMF to have low levels of root colonisation when available P is extremely low (Bolan et al. 1987; Jeffery et al. 2017, 2018), we did not observe this in the most P-limited stages. Hence, we surmise that albeit P-poor, the Warren chronosequence does not exhibit P levels low enough to inhibit AMF (Jeffery et al. 2018; Albornoz et al. 2021).

Under the microscope, root colonisation by G-AMF was observed in both plant species, but no M-AMF were observed in *M. stipoides*. Nevertheless, M-AMF were found in the DNA analysis of *M. stipoide*s roots and the two most abundant OTUs of M-AMF were found in both plant species. This indicates that M-AMF were indeed present in the native grass. It is possible that colonisation was so low that it was not observed under the microscope in the subset of the root system that was mounted on slides and assessed. It also is possible that staining methods were not appropriate for identifying M-AMF in the roots of *M. stipoides* despite their presence. Native plant species to south-Western Australia have developed a wide range of root adaptations that might have interfered with the staining process. Staining artefacts can sometimes occur as a by-product of specific interactions between AMF and host roots (Gange et al. 1999; Dodd et al. 2000) and it is possible that the M-AMF in our study eluded microscopic observation in this way. *Microlaena stipoides* roots have not been stained for M-AMF before this study and, while staining methods were appropriate for G-AMF, alternative staining methods may be required to observe M-AMF in this grass such as those outlined in Phillips and Hayman (1970), Brundrett (1994), or Koske and Gemma (1989).

Watering treatments differently influenced root colonisation by M-AMF and G-AMF within the exotic host *T. subterraneum*. Mucoromycotinian AMF showed the highest colonisation only in the wet treatment of Stage 1. We expected root colonisation by M-AMF to not be affected by watering treatments given M-AMF apparent flexibility regarding water availability, with high colonisation often correlating with extreme water availability conditions (Staddon et al. 2004; Orchard et al. 2016). While this was not the case in our study, we saw an inconsistent influence of water treatments on root colonisation by M-AMF, with no effect at most stages. This suggests that other soil factors such as nutrient availability, which showed more consistent effects along the soil chronosequence, or plant-fungal interactions, were more important influences on root colonisation by M-AMF. Drought stress to plants can decrease C supply to mycorrhizal roots (Wang et al. 2021), which may in turn influence fungal root colonisation. However, we saw an increase in G-AMF root colonisation in water-stressed *T. subterranean*, and no influence in *M. stipoides*, suggesting that our dry watering treatment may not have stressed plants enough to reduce C deposition to roots.

As we observed no root colonisation by M-AMF of *M. stipoides* under the microscope, it remains ambiguous how water availability affects root colonisation in this species. In contrast, root colonisation by G-AMF was higher in the dry treatment of all stages in the exotic host *T. subterranean.* Glomeromycotinian AMF can improve drought resistance in hosts by scavenging for water and regulating soil moisture around plant roots (Wu and Zhou 2017). Glomeromycotinian AMF are found in native systems throughout Australia (Albornoz et al. 2022); however, research is needed to understand the extent that native Australian plants rely on their mycorrhizas. The declining rainfall and increasing evidence of water stressed ecosystems within south-west Western Australia (Evans et al. 2013), coupled with the importance of rainfall to both types of fungi (Albornoz et al. 2022), deems this a worthy topic of further research.

### M-AMF and G-AMF communities shift along the retrogressive soil chronosequence

Our DNA results showed clear succession of communities of G-AMF from the youngest to oldest chronosequence stage, whereas communities of M-AMF were distinct in the youngest and oldest stages but did not differ between intermediate stages. The full diversity of M-AMF is still yet to be sequenced and added to publicly available libraries, so our study could only determine M-AMF taxa to Endogonaceae. Nonetheless, the most abundant OTU of M-AMF was dominant, but not exclusive, in the youngest stage, Stage 1 (OTU 6), while the second most abundant was primarily in the oldest stage, Stage 6 (OTU 66) and did not occur in Stage 1. This suggests ecological niche differentiation within M-AMF that could be driven by soil and vegetation properties that change during ecosystem development. The same also was apparent for G-AMF, with the most abundant OTU (OTU 2) predominantly in the most acidic and P-limited Stage 6. This OTU was from the Acaulosporaceae family which are suggested to be stress-tolerators (Chagnon et al. 2013) that can often survive in a low pH environments (e.g., Porter et al. 1987; Morton 1986; Palenzuela et al. 2013). This could mean that M-AMF OTU 66 similarly is a stress-tolerator, as this dominant OTU was only present in the most acidic and P-limited stage. Additionally, different assemblages of both fungi were recorded on the two plant hosts, including taxa unique to one or other that suggests specialised host-fungi relationships. This is noteworthy for G-AMF as their host-range is currently unresolved, and families of G-AMF can have different levels of host-specificity (Zheng et al. 2016). Further investigation of the taxonomy and environmental drivers of M-AMF is needed to understand potential niche specialisations within the M-AMF.

Richness of M-AMF was inversely associated with soil P, while richness of G-AMF followed the opposite trend, suggesting ecological niche differences between the fungus types. Harsh environments are sometimes found to select for M-AMF rather than G-AMF (Wang et al. 1993; Orchard et al. 2017b), such as in a low pH environments where they can replace G-AMF (Postma et al. 2007; Göransson et al. 2008). These findings are consistent with our results, as M-AMF richness was the highest in the most acidic and P-limiting the oldest Stage 6, and second highest in the P-limited youngest Stage 1. In contrast, richness of G-AMF was the highest in the most P-available Stage 3, but low in both P-limited stages. Hence, it is likely that the G-AMF present in the oldest and youngest stages were a subset of species adapted to an extremely low P (such as Acaulosporaceae; Chagnon et al. 2013). Mucoromycotinian AMF showed preference for these stages, suggesting M-AMF have similar stress tolerance to highly P-limited, acidic environments. These findings provide more support for distinct ecological niches for M-AMF and G-AMF (Albornoz et al. 2021).

Plant communities influence richness of M-AMF and G-AMF (Krüger et al. 2015). While we did not include plant community in our experimental design, it is closely linked to soil age and therefore indirectly included in our study. In another retrogressive soil chronosequence in Jurien Bay, south-western Australia, Zemunik et al. (2015) found that arbuscular mycorrhizal (AM) plant species cover declines with decreasing P-availability as alternative nutrient-acquisition strategies such as cluster roots (Lambers et al. 2017) or saprophytic fungi (Balser et al. 2005) become dominant (Lambers et al. 2008). Our findings that richness of G-AMF declined with soil age could be because of a decline in AM plant species cover, but we found the opposite pattern for richness of M-AMF. It is also possible that AM plant species richness was influential to M-AMF and G-AMF richness; however, Krüger et al. (2015) found G-AMF richness to not follow AM plant species richness along the Jurien Bay soil chronosequence. Clearly the explanation is not as simple as plant cover or diversity above-ground matching fungal richness belowground. Disentangling the ‘black box’ of microbial interactions could help to understand these linkages (Albornoz et al. 2022).

## Conclusions

The soil chronosequence provided an ideal system to test ideas about the interactive effects of key factors on G-AMF and M-AMF while holding soil origin and dispersal limitations constant. Our results show that communities of M-AMF and G-AMF occupy distinct ecological niches along a retrogressive soil chronosequence but have similar anatomical root colonisation patterns. Under low levels of available soil P, root colonisation by both fungi seemed to correlate with soil P but not soil pH. This was surprising as root colonisation by M-AMF can be strongly influenced by soil pH (Postma et al. 2007; Göransson et al. 2008). Nonetheless, richness of M-AMF was the highest in the most acidic, P-limited stage, supporting claims that M-AMF are best suited to harsh environments (Wang et al. 1993; Orchard et al. 2017b; Albornoz et al. 2022). Additionally, root colonisation by M-AMF and G-AMF in *T. subterraneum* showed different responses to wet and dry treatments, further supporting suggestions of ecological niche differentiation between the fungi. Preferences of G-AMF for the dry treatment confirms evidence of drought tolerance for these fungi (Wu and Zhou 2017), although this was only evident in the exotic host. Alternatively, richness of M-AMF showed some, albeit limited, preference for the wet treatment contrary to predictions that M-AMF would not be affected owing to broad niches with respect to water availability (Staddon et al. 2004; Orchard et al. 2016). However, the lack of a consistent response to the wet treatment along the soil chronosequence suggests that nutrients or other factors were more important than our moisture regimes. The global distribution of both G-AMF and M-AMF (Brundrett 2009; Kivlin et al. 2011; Orchard et al. 2017b), despite their absence from some biomes (Albornoz et al. 2022), emphasises the importance of further research into their ecology and evolution. In particular, the ready association of M-AMF with an exotic agricultural host plant suggests a need for further investigation of their role in agroecosystems.

## Supplementary Information

Below is the link to the electronic supplementary material.

Supplementary file1 (PDF 163 KB)

Supplementary file2 (PDF 287 KB)

Supplementary file3 (DOCX 4524 KB)

## Data Availability

The data and R code generated in this study are available from the corresponding author upon reasonable request. DNA sequences will be made available on NCBI upon paper acceptance.
