# Supplementary material for: Niche differentiation of Mucoromycotinian and Glomeromycotinian arbuscular mycorrhizal fungi along a 2-million-year soil chronosequence
Source: Mycorrhiza. 2023 May 11;33(3):139–52. doi: 10.1007/s00572-023-01111-x (PMC10244280; doi:10.1007/s00572-023-01111-x)
Supplement: Supplementary file 1 — Supplementary file1 (PDF 163 KB) [file 572_2023_1111_MOESM1_ESM.pdf]

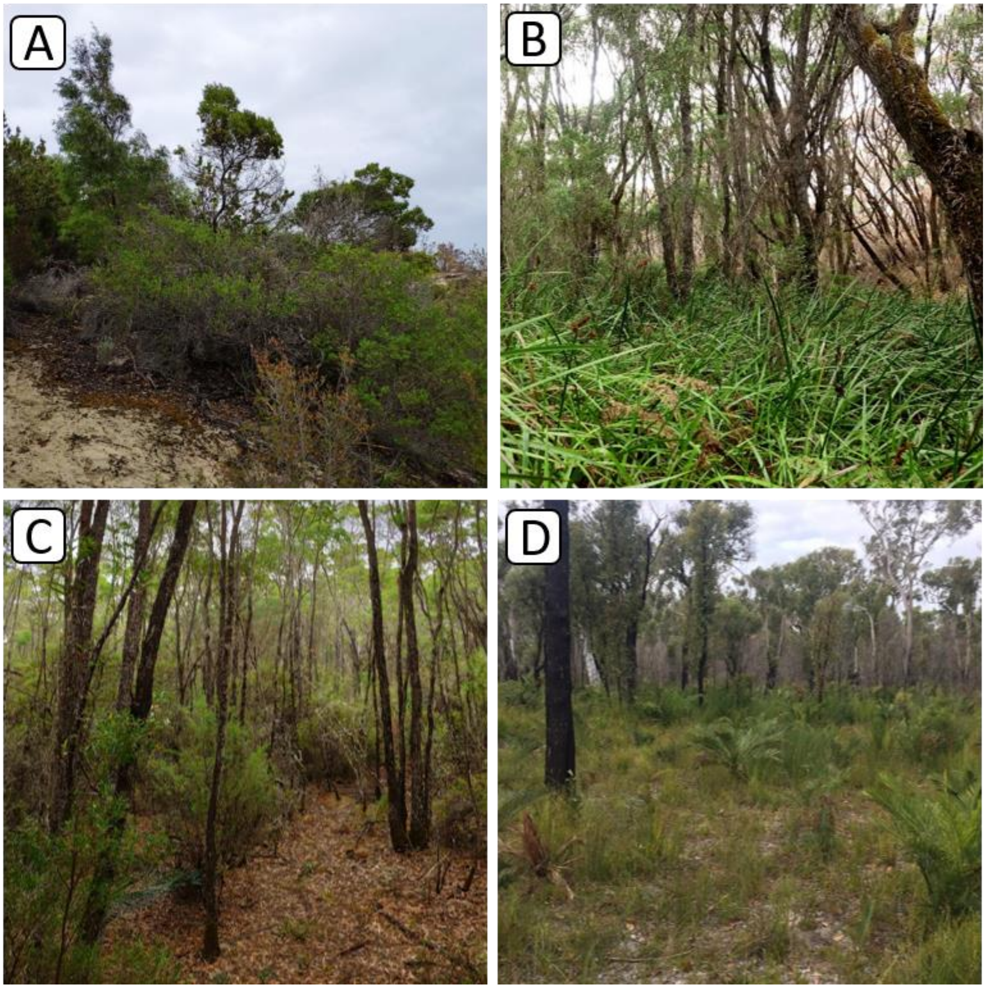


**Figure S1.** Four stages, in ascending chronosequence stage, of the Warren soil chronosequence in south-west Western Australia. This soil chronosequence is a sequence of dunes generated over 2-million years. Stage 1 (A) Meerup Leached over Calcerous Sands <6.5 ka. Stage 2 (B) Meerup Podzols over Calcerous Sands ~6.5 ka. Stage 3 (C) Meerup Podzols in Silicerous Sands 120-500 ka. Stage 4 (D) Cleave >2,000 ka. Dune classification follows Turner et al. (2018). These sites all follow the Warren Beach Road, south of the Warren River. Images: Ruipeng Yu.
