# Supplementary material for: Niche differentiation of Mucoromycotinian and Glomeromycotinian arbuscular mycorrhizal fungi along a 2-million-year soil chronosequence
Source: Mycorrhiza. 2023 May 11;33(3):139–52. doi: 10.1007/s00572-023-01111-x (PMC10244280; doi:10.1007/s00572-023-01111-x)
Supplement: Supplementary file 2 — Supplementary file2 (PDF 287 KB) [file 572_2023_1111_MOESM2_ESM.pdf]

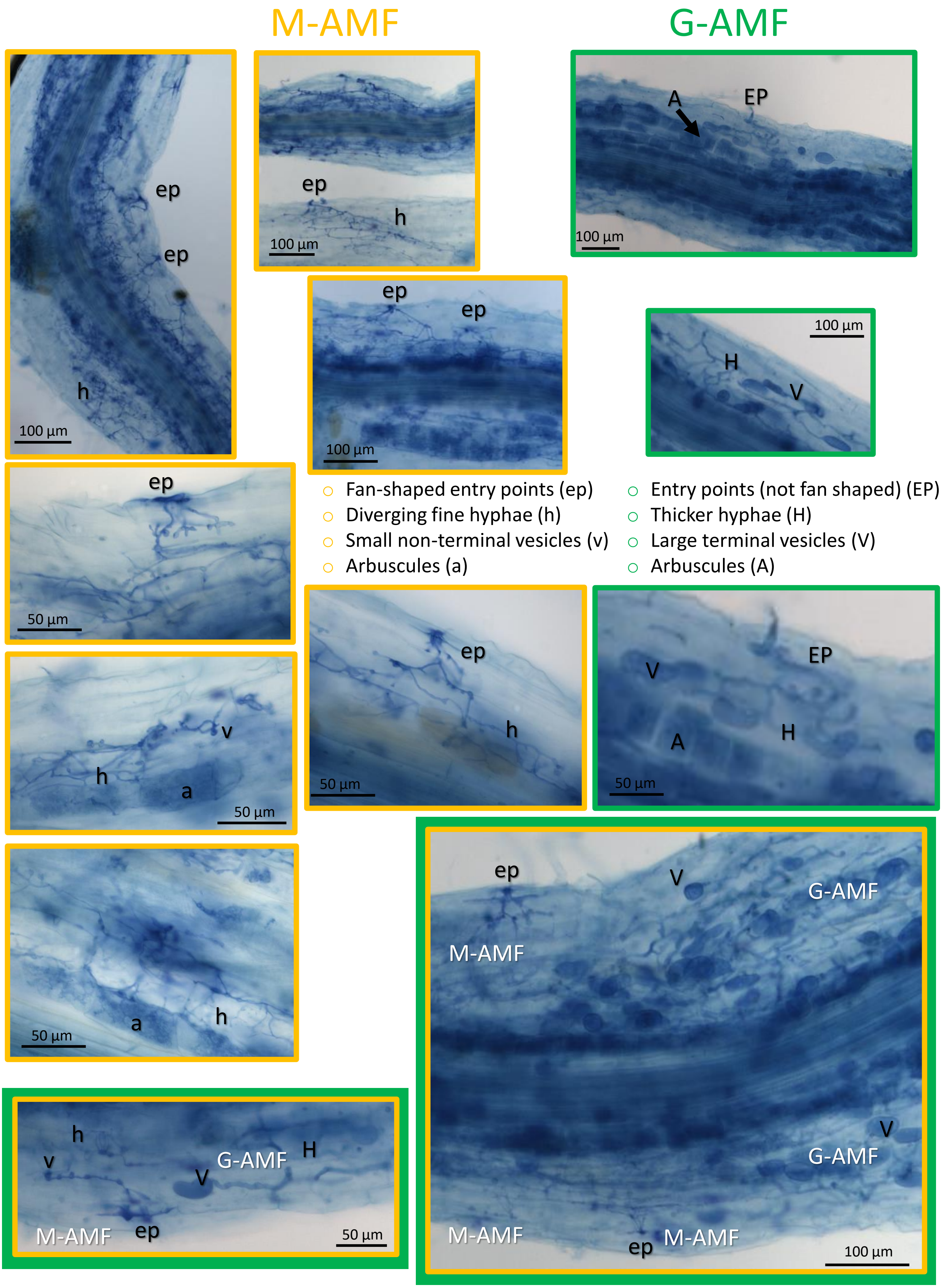


**Figure S2.** The distinctive features of Mucoromycotinian-AMF (M-AMF; yellow outlines) and Glomeromycotinian-AMF (G-AMF; green outlines) in stained roots of *Lotus tenuis*. This figure was used as a reference when assessing the percentage of root length colonised by M-AMF and G-AMF (photographs by Jeremy Bougoure).
