# Supplementary material for: Niche differentiation of Mucoromycotinian and Glomeromycotinian arbuscular mycorrhizal fungi along a 2-million-year soil chronosequence
Source: Mycorrhiza. 2023 May 11;33(3):139–52. doi: 10.1007/s00572-023-01111-x (PMC10244280; doi:10.1007/s00572-023-01111-x)
Supplement: Supplementary file 3 — Supplementary file3 (DOCX 4524 KB) [file 572_2023_1111_MOESM3_ESM.docx]

## **Niche differentiation of Mucoromycotinian and Glomeromycotinian arbuscular mycorrhizal fungi along a 2-million-year soil chronosequence**

Thomas M Mansfield^1*^, Felipe E. Albornoz^1,2,3^, Megan H. Ryan^3^, Gary D. Bending^4^ and Rachel J. Standish^1^

^1^Environmental and Conservation Sciences, Murdoch University, Murdoch, WA 6150, Australia.

^2^ Commonwealth Scientific and Industrial Research Organisation, Land and Water, Wembley, WA, Australia

^3^UWA School of Agriculture and Environment, University of Western Australia, Crawley, WA 6009, Australia.

^4^School of Life Sciences, University of Warwick, Coventry, CV4 7AL, United Kingdom.

*Corresponding Author: Thomas Mansfield (Ph: +61 452 575 312; email: Thomas.Mansfield@murdoch.edu.au; postal address: 90 South Street, Murdoch University, Murdoch, WA, 6150, Australia)

Journal applying to: Mycorrhiza

## **Supplementary Figures**


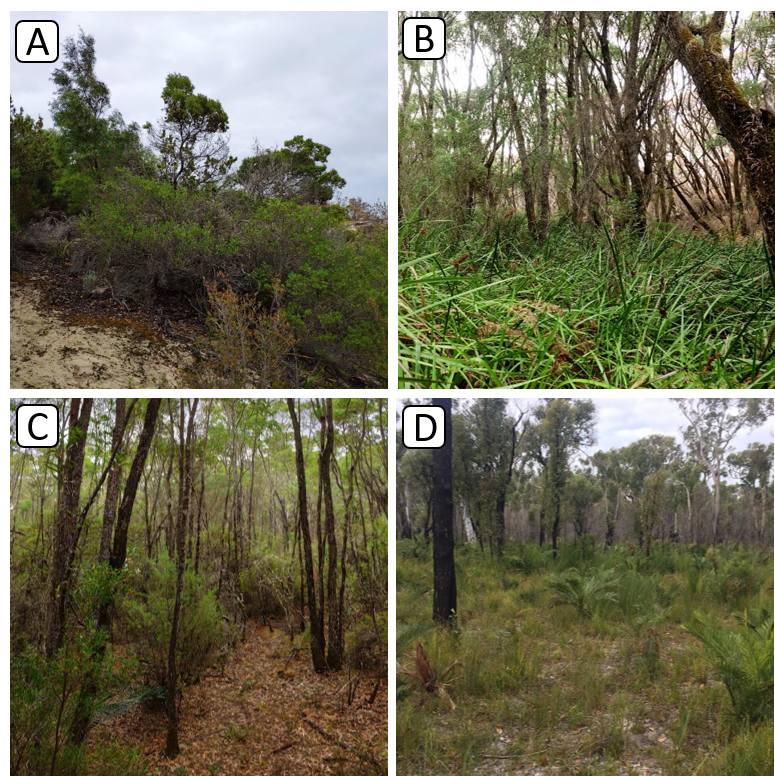


**Figure S1.** Four stages, in ascending chronosequence stage, of the Warren soil chronosequence in south-west Western Australia. This soil chronosequence is a sequence of dunes generated over 2-million years. Stage 1 (A) Meerup Leached over Calcerous Sands <6.5 ka. Stage 2 (B) Meerup Podzols over Calcerous Sands ~6.5 ka. Stage 3 (C) Meerup Podzols in Silicerous Sands 120-500 ka. Stage 4 (D) Cleave >2,000 ka. Dune classification follows Turner et al. (2018). These sites all follow the Warren Beach Road, south of the Warren River. Images: Ruipeng Yu.


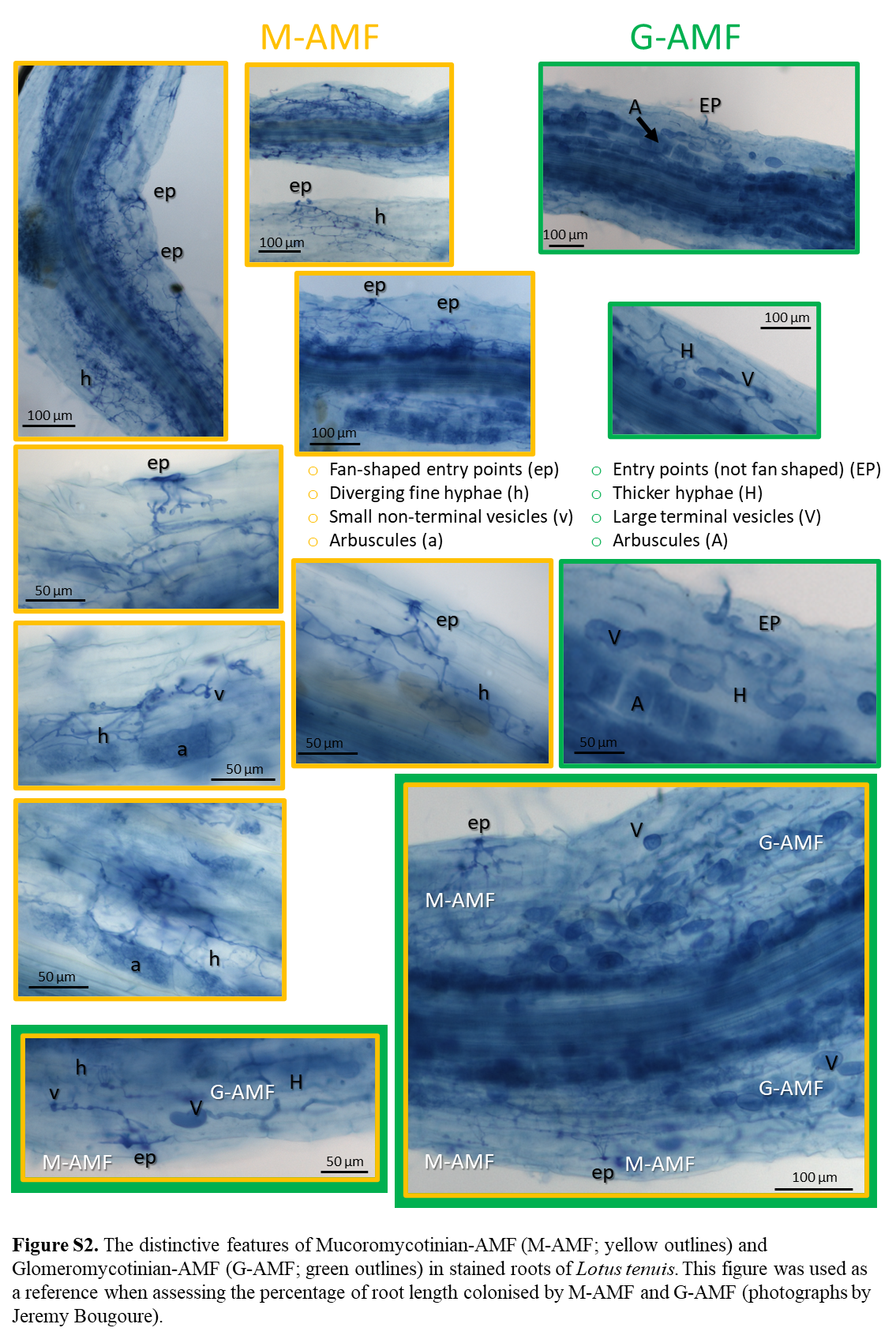


**Figure S2.** The distinctive features of Mucoromycotinian-AMF (M-AMF; yellow outlines) and Glomeromycotinian-AMF (G-AMF; green outlines) in stained roots of *Lotus tenuis*. This figure was used as a reference when assessing the percentage of root length colonised by M-AMF and G-AMF (photographs by Jeremy Bougoure).
